# Long Non-Coding RNA and Epigenetic Gene Regulation of KSHV

**DOI:** 10.3390/v6114165

**Published:** 2014-11-04

**Authors:** Mel Campbell, Hsing-Jien Kung, Yoshihiro Izumiya

**Affiliations:** 1 Department of Dermatology, University of California, Davis, CA 95616, USA; E-Mail: yizumiya@ucdavis.edu; 2 UC Davis Comprehensive Cancer Center, University of California, Davis, CA 95616, USA; E-Mail: hkung@ucdavis.edu; 3 Department of Biochemistry and Molecular Medicine, University of California, Davis, CA 95616, USA; 4 National Health Research Institutes, Maioli 35053, Taiwan; 5 Taipei Medical University, Taipei 110, Taiwan

**Keywords:** viruses, long non-coding RNA, epigenetics, viral latency, viral reactivation

## Abstract

Kaposi’s sarcoma-associated herpesvirus (KSHV/human herpesvirus 8) is a γ-herpesvirus linked to Kaposi’s sarcoma (KS) and two lymphoproliferative disorders, primary effusion lymphoma (PEL or body-cavity B-lymphoma [BCBL]) and a subset of Multicentric Castleman’s Disease. During lytic growth, pervasive viral transcription generating a variety of transcripts with uncertain protein-coding potential has been described on a genome-wide scale in β- and γ-herpesviruses. One class of such RNAs is called long non-coding RNAs (lncRNAs). KSHV encodes a viral lncRNA known as polyadenylated nuclear RNA (PAN RNA), a copious early gene product. PAN RNA has been implicated in KSHV gene expression, replication, and immune modulation. PAN RNA expression is required for optimal expression of the entire KSHV lytic gene expression program. Latent KSHV episomes are coated with viral latency-associated nuclear antigen (LANA). LANA rapidly dissociates from episomes during reactivation. Here we review recent studies suggesting that PAN RNA may function as a viral lncRNA, including a role in the facilitation of LANA-episomal dissociation during lytic replication.

## 1. Introduction

Recent new developments in epigenetic control of transcriptional programs by chromatin modifiers are highlighted by findings that long non-coding RNA (lncRNA), previously thought to be “transcription noise”, interact with chromatin-associated proteins to modulate their functions [1,2]. lncRNA interactions have been reported with most classes of proteins that associate with chromatin including transcription factors [3], chromatin remodelers [4], and histone methylases and demethylases [5,6]. Non-coding (nc) RNAs can be classified as either housekeeping or regulatory ncRNA, and based on transcript size, regulatory ncRNA can be further grouped into two subclasses; small non-coding RNA (20–200 nt) and long non-coding RNA (lncRNA, >200 nt). Although the role of small regulatory RNAs (microRNAs and siRNAs [small interfering RNA]) in gene silencing is well defined, the biological function of lncRNAs is still unclear. Like proteins, diverse biological functions of lncRNAs have been identified/proposed, including the structural integrity of the nucleus, regulation of gene expression, chromatin remodeling, transcription, and posttranscriptional processing [7,8]. The actions of several lncRNAs and their mechanisms are summarized in the Table 1.

**Table 1:** Mechanisms of lncRNA action.

Name of lncRNA	Nature of lncRNA/Mode of Action	Consequence of the Regulation	Reference
ANRIL, HOTAIR, XIST, H19, KCNQ1OT1	Scaffold molecule/Histone modification	Epigenetic gene silencing	[6,9,10,11,12]
NEAT1	Scaffold molecule/Protein assembly	Paraspeckles formation	[13]
MALAT1	Modifier of alternative splicing/Relocation of splicing factor	Modulation of alternative splicing	[14]
BACE1AS	Regulation of mRNA stability/Modification of mRNA stability	Increased translation of BACE1	[15]
Pseudo-NOS	Translational control/Displacement of ribosome	Repressed translation of nNOS	[16]
GAS5	Decoy for DNA binding/Inhibition of DNA binding	Repressed GR mediated gene activation	[17]

Currently, the most dominant function explored in lncRNA studies relates to epigenetic regulation of target genes. This role is typically associated with gene repression, which has been studied in many cellular lncRNAs including ANRIL, HOTAIR, H19, and XIST [6,9,10,11,12]. These lncRNAs exhibit their repression function through interactions with histone modifying enzymes. The most common protein partners of lncRNAs are the PRC1 and PRC2 polycomb repressive complexes. These complexes transfer repressive posttranslational modification marks (H2AK119ub or H3K27me3) to histone tails, thereby facilitating chromatin compaction to repress gene transcription [1]. It is estimated that nearly 20% of all lncRNAs bind PRC2 [18], although the biological meaning of this observation remains unclear. ANRIL, HOTAIR, H19, KCNQ1OT1, and XIST have been shown to interact with the PRC2 complex, and in all except H19, direct binding has been observed between PRC2 proteins and the lncRNAs [6,9,10,11,12], reviewed in [19]. The programmers of chromatin remodeling are enzymes involved in histone modifications, namely histone methylases and demethylases, thus regulation of such enzymes by lncRNAs through recruitment or assembly of specific complexes may have profound local effects on chromatin modification.

## 2. KSHV Epigenetics

While the KSHV genome is histone-free in virions, the viral genome adopts a highly organized chromatin structure in infected cells that have established latency [20,21] and following *de novo* infection [22]. During *de novo* infection there is a rapid association of KSHV genomic DNA with histones, followed by a biphasic period of chromatinization [22]. These early events include an initial transient enrichment of H3K4me3 and H3K27ac activating histone marks on the viral chromatin that is followed by a decline of activating marks and the transition to a heterochromatic state enriched for H3K27me3/H2AK119ub marks. This transition is dependent on PRC1 and PRC2 complexes and ultimately results in the inhibition of lytic gene expression, however, these events differ among cell types [22]. During latency, the promoter of the master lytic switch regulator, K-Rta, is characterized by a bivalent chromatin structure, consisting of both activating and repressive histone marks, including H3K27me3 [20,21]. Upon reactivation, immediate early/early gene expression, including K-Rta, is accompanied by decreased local levels of repressive H3K27me3 marks while activating histone marks such as acetylated H3 and H3K4me3 are simultaneously increased. This shift is important for optimal K‑Rta production and activation of the entire KSHV lytic program [20,21]. In all of these studies [20,21,22], the deposition of histone marks on viral chromatin is site-specific, with certain histone marks enriched only on specific viral genomic regions. Although the exact mechanisms of targeted recruitment of histone-modifying machinery are unclear, it is likely that cellular and viral DNA-binding factors may be important for recruitment [22]. In addition, the lncRNA function of PAN RNA has been suggested to be important in this regard, perhaps serving as a guide RNA to deliver factors to specific viral genomic locations [23] (see below). We have been interested in how viral gene products can change the epigenetic landscape of the viral and host genome. We, and others, have found that cellular histone lysine methyltransferases (KMT), protein arginine methyltransferases (PRMT), and lysine demethylases (KDM) are targets of viral proteins and important for the viral life cycle [21,24,25,26,27,28]. The potential significance of histone methylases for KSHV biology was also highlighted by the recent finding that histone modifying enzymes, but not modified histones *per se*, act as epigenetic marks for inheritance [29]. In the study, Petruk *et al.* showed that methylated histones were not detected during S-phase of the cell cycle, however Trithorax and Ezh2, which are H3K4 and H3K27 methylases were continuously associated with their response elements on the newly replicated DNA which thus re‑establishes the histone marks on newly assembled unmethylated histones. This finding suggests that dislodging PRC1/PRC2 polycomb complexes from the KSHV genome may be important for effective viral replication, because the KSHV genome has been shown to be heavily loaded by polycomb complexes [20,21,22]. This further supports the notion that removal of enzymatic complexes that deposit or maintain a repressive KSHV chromatin state is required for optimal expression of the lytic program. As deletion studies have suggested a generalized repressive role for LANA in lytic gene expression [30], it is tempting to speculate that sequestering polycomb complexes and/or LANA from KSHV genomes is important to allow late gene expression. Persistence of these complexes might allow them to bind newly synthesized DNA [29] and continue to silence viral gene expression. In the following sections, we propose that one of the lncRNA-like functions of PAN RNA may be in the titration of the repressive complexes away from the KSHV genome during reactivation.

## 3. KSHV PAN RNA

KSHV encodes a 1.1 kb viral lncRNA known as polyadenylated nuclear RNA or PAN RNA, an abundant early gene product. High level expression of PAN RNA is directly regulated by a master switch gene K-Rta (replication and transcription activator [31,32,33]. While PAN RNA was first described 18 years ago [31,34,35] as a non-coding RNA, its discovery predated the widespread recognition of long non-coding RNAs and their role in gene regulation. The structure, unique stability, and nuclear retention properties of PAN RNA has been exquisitely characterized by the groups of Conrad, Steitz, and Zheng [36,37,38,39,40,41]. Despite this knowledge, the role of PAN RNA in KSHV lifecycle is still unclear. New studies by Steitz, Pari and colleagues have begun to address this question, and PAN RNA has been demonstrated to play a role in KSHV gene expression, replication, and immune modulation [23,42,43,44]. With the recent discovery of thousands of lncRNAs, PAN RNA has been re-examined under the premise that one of its functions may be that of a viral lncRNA. Recent reports have demonstrated that PAN RNA binds the transcription factor IRF4 [43], the lysine demethylase JMJD3 [44], and the lysine methylase Ezh2, [23] supporting the notion that similarly to cellular lncRNAs, PAN RNA may function in epigenetic gene regulation. Although PAN RNA is classified as a lytic transcript, prior reports have demonstrated its presence in virions [45] and virion packaging has been further demonstrated by others [23]. Moreover, recent studies have also reported that PAN RNA is expressed in uninduced KSHV positive cell lines [23,46] suggesting that PAN RNA is a transcript present (at variable levels) during both latency and lytic infection. Thus, the current view of PAN RNA function depicts broad effects of PAN RNA expression acting as a multifunctional regulator, which influences both viral and host transcriptional programs.

### 3.1. LANA Is Released from KSHV Genome in a Binding Site-Specific Manner

Our discovery of PAN RNA involvement in the regulation of LANA began with the observation that LANA is released from the KSHV genome immediately after triggering reactivation by K-Rta expression [25]. For the study, we took four different time points to examine the dynamics of LANA recruitment sites on the KSHV genome. Initially, we expected that LANA binds to different sites during the course of reactivation as LANA was reported to interact with K-Rta [47]. Unexpectedly, we found that LANA is dissociated from the KSHV genome almost immediately after induction of lytic replication by K-Rta expression. In our experiments, we used K-Rta inducible cells instead of chemicals to synchronize and induce viral lytic replication in almost all cells [48]; this strategy significantly reduced the background caused by non-reactivating cells. Accordingly, the negative effects (reduction of recruitment) could be observed. Importantly, dissociation of LANA is only seen along the unique region of the KSHV genome, and not at the terminal repeat region (TR), where LANA was still enriched nearly 40-fold over input DNA in our tiling array analyses at all time points [25].This may be due to the combination of tightness of LANA binding to the TR region through the DNA binding domain as well as copy numbers of TR elements in the infected cells. Dissociation of LANA after reactivation was further confirmed with independent ChIP experiments with qt-PCR [49]. As LANA is generally viewed as a key factor for maintaining latency, sequestration of LANA from the KSHV genome may play a role in regulating lytic gene expression. Ganem and colleagues have also reported that the association of LANA with viral chromatin is disrupted in cells during the lytic cycle [50].

### 3.2. PAN RNA Interacts with LANA

What molecular mechanisms would underlie LANA dissociation from KSHV episomes? As our lab and another group independently found; LANA associates with multiple RNA-binding proteins *in vivo* [26,51]. We hypothesized that LANA might possess the property of RNA binding and we speculated that robust expression of PAN RNA might have a role in dissociation of LANA during lytic replication. In fact, interactions between lncRNAs and transcriptional factors have been reported elsewhere [3]. Could PAN RNA function in an lncRNA-like manner to reduce LANA’s occupancy on chromatin by acting as a protein sponge? To test the hypothesis, a series of experiments were conducted to examine if LANA associates with PAN RNA *in vitro* and *in vivo*. The results clearly demonstrated that LANA has a property to bind PAN RNA [49]. LANA may recognize RNA secondary structure in addition to sequence as several experiments using different salt concentrations repeatedly showed that antisense PAN RNA also bound to LANA, albeit weaker than PAN RNA in the sense orientation [49]. Subsequent mapping experiments with a series of PAN RNA deletions showed that the nucleotides towards the 3' end of PAN RNA are responsible for the interaction [49]. By using RNA immunoprecipitation (RIP) analyses with anti-LANA antibody, LANA was shown to be associated with PAN RNA in naturally infected cells during the course of reactivation [49]. The RNA-binding domain of LANA was mapped with both RNA- and GST-pull-down analyses. Intriguingly, results showed that the RNA-binding domain completely overlapped with the previously identified LANA histone-binding domain (residues 1–20) [49]; this may be the mechanistic explanation of why LANA dissociates from the unique region, but not TR region of the KSHV chromosome during reactivation, as TR binding requires a DNA-binding domain, located at the C‑terminal region of LANA [52,53]. It is exciting to speculate that PAN RNA may have evolved as a viral “Aptamer” for LANA to counteract its function and allow for effective lytic replication.

### 3.3. PAN RNA Is Responsible for LANA Dissociation from KSHV Genomes

To study if PAN RNA/LANA association is responsible for dissociation of LANA from KSHV genomes, we adapted the strategy reported by the Steitz lab [42] which utilizes modified antisense oligonucleotides and endogenous RNase H to knock-down PAN RNA expression during reactivation. Similar to their research design, we also designed antisense oligonucleotides for the K7 transcript as a control, because K7 partially overlaps with PAN RNA and co-terminates with PAN RNA transcripts. The antisense oligonucleotides were electroporated into latent KSHV positive cell lines prior to reactivation. The effects on LANA binding to the KSHV genome by the partial ablation of PAN RNA expression were then examined by ChIP assay with anti-LANA antibody. The results showed that knocking-down of PAN RNA expression during reactivation resulted in retained LANA occupancy on the KSHV genome [49]. This results support the idea that PAN RNA plays a role in the sequestration of the LANA complex from the KSHV genome; however, how binding of LANA on the KSHV chromosome affects the landscape of KSHV histone modifications remains to be seen. Thus, one function of the lytic lncRNA PAN RNA includes an influence on the activity of a latent viral DNA-binding protein (LANA). Partial inhibition of PAN RNA production has been shown to decrease late gene expression significantly [42]. PRC2 complex components interact with both PAN RNA and purified LANA *in vitro* [61] and *in vivo* [23]. Together, these results suggest that PAN RNA is important for optimal lytic gene expression, in part through mechanisms involving sequestration of repressive protein complexes away from KSHV genomes undergoing reactivation.

### 3.4. Other Functions of PAN RNA

PAN RNA has been reported to interact with both viral and cellular proteins suggestive of a multifunctional role for PAN RNA in the KSHV lifecycle. This interaction list includes KSHV ORFs 26, 57, and 59 [40,41,43,54,55], as well as host-encoded factors such as histones, single-stranded DNA binding proteins, transcription factors, ribonucleoproteins, and histone methylases and demethylases [23,40,42,43,44]. Follow up studies have been performed for several of these interactions. PAN RNA interaction with both ORF57 and the cellular ribonucleoprotein PAPBC1 has been reported to be involved in the abundant nuclear accumulation of PAN RNA [40,42,54]. PAN RNA interaction with the transcription factor IRF4 [43], the histone demethylases UTX and JMJD3, the histone methylase MLL2 [44], and components of the polycomb complex 2 (PRC2) [23], have suggested that PAN RNA has potential widespread effects on both KSHV and cellular gene expression and epigenetic states. Moreover, using chromatin isolation by RNA purification (ChIRP) analysis [56], Rossetto *et al*. [23] have shown PAN RNA interacts with many regions of both the KSHV genome and cellular chromosomes. These interactions influence almost the entire KSHV transcriptional program as well as host genes controlling the cell cycle, inflammation, and immune responses. Finally, similar to what has been previously observed with cellular lncRNAs [57], a recent ribosomal profiling study of KSHV infected cells has found a fraction of PAN RNA to be associated with ribosomes, suggesting that in addition to its role as a non-coding RNA, PAN may also serve as a *bona fide* mRNA with the potential to produce several small viral peptides [58].

## 4. Concluding Remarks

**Figure 1:**
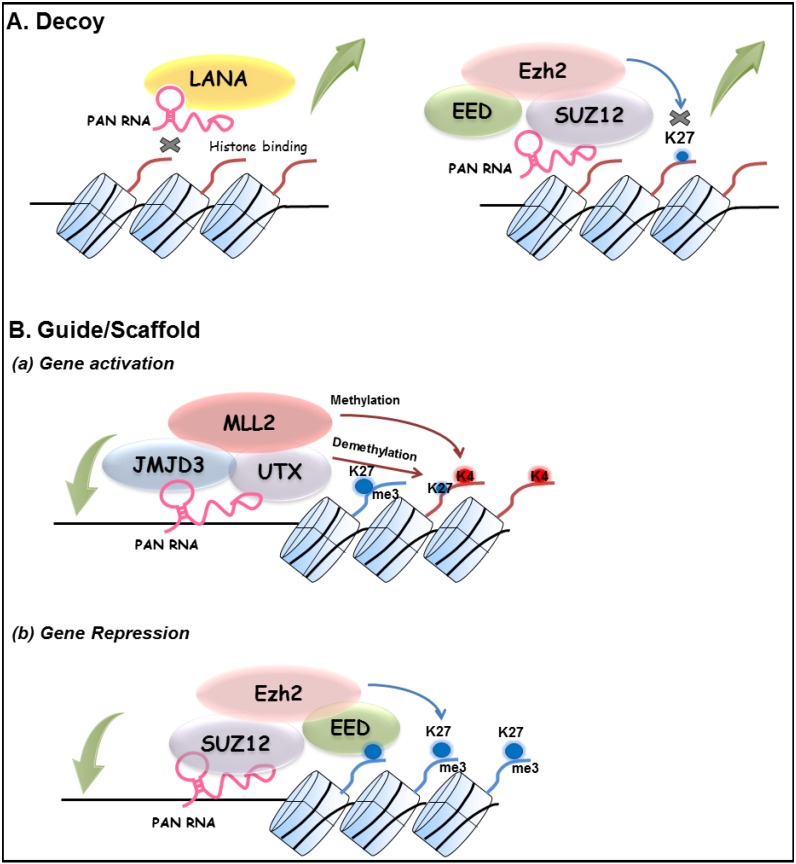
Working model of KSHV PAN lncRNA action. (**A**) Decoy. Expression of PAN RNA sequesters LANA (left) and chromatin modifying complexes such as PRC2 (right) from the KSHV genome that reduces or prevents H3K27me3 mark deposition [1]. (**B**) Guide/Scaffold. PAN RNA aids in the targeting of regulatory factor complexes to specific loci of viral or cellular genes, which regulate gene expression to suit viral needs requiring (**a**) activation (*i.e.*, ORF50 locus during reactivation) (**b**) repression (*i.e.*, cellular response to viral infection or reactivation [23,43,44]. Mechanisms of PAN RNA targeting to specific loci is currently unknown. Not shown is the potential for PAN RNA to act in *cis* as a lncRNA signal [1]. Large curved green arrows indicate complex eviction (upward arrow) or deposition (downward arrow) from a genomic locus. Histones are depicted as cylinders with histone tails emanating, (red tail, activation; blue tail, repression) K27 (demethylated histone H3K27) and K27me3 (histone H3K27 tri-methylated; repressive mark); K4 (histone H3K4, substrate for methylation, activating mark).

Pathogen-encoded lncRNAs have been reported for several virus groups, including herpesviruses (see [59] for a recent review). For KSHV, several studies have described properties of PAN RNA, which are consistent with its role as a multifunctional viral lncRNA. In view of the dependence of viral replication on the host cell machinery, it is perhaps not surprising that viral and cellular lncRNAs are increasingly linked to viral processes. It should be noted that our studies have focused on PAN RNA as a molecular sink or decoy for LANA or other repressive protein complexes in order to facilitate lytic replication [49]. In addition to that aspect of the model (Figure 1), the results of Rossetto, Pari and colleagues [23,43,44] suggests that PAN RNA is capable of functioning in a lncRNA-like manner using other archetypes of lncRNA molecular mechanisms as put forth by Wang and Chang, including lncRNA guides, scaffolds and signals [1]. For example, the widespread distribution of PAN RNA at both KSHV and cellular genomic loci suggests that PAN RNA may act as a guide or scaffold to target chromatin-modifying complexes to specific locations with PAN RNA serving as a conduit for either gene activation or repression [23,44]. This paradigm is illustrated by the detection of PAN RNA interactions at the ORF50 promoter and with JMJD3, UTX, and MLL2; these interactions are consistent with the changes in local histone modifications observed at this locus during viral reactivation [20,21]. Interestingly, a recombinant bacmid lacking PAN RNA expression does not produce virus, nor could viral production by this mutant be rescued by K-Rta overexpression [23]. Although PAN RNA is considered a K-Rta target gene [32,33] these surprising results indicate that a certain level of PAN RNA is needed for the activation of the entire KSHV lytic expression program. While PAN RNA is often considered a lytic transcript, it is important to keep in mind that PAN RNA is packaged into virions [45] and the transcript is also detected in latent cells [23], Thus PAN RNA has the potential to influence virus-host interactions at all stages of infection. One curious aspect that arises when attempting to invoke lncRNA mechanisms for PAN RNA concerns the extreme abundance of the transcript. Early studies had suggested a number of up to ~500,000 copies of PAN RNA per induced BC-1 cell representing ~80% of the total poly(A) selected RNA pool [31,32]. This abundance has been confirmed by next generation mRNA-sequencing results where PAN RNA represented up to 90% of the KSHV reads at 72 h post-induction [58]. This expression level contrasts that of cellular noncoding RNAs, which have been reported to often be expressed at significantly lower levels than coding RNA [60]. While one could propose that this high level of PAN RNA expression (a) creates a better sponge or decoy; (b) is necessary to ensure its incorporation into virions; or (c) assures perturbation of host cell transcriptional programs, it will be exciting to see how specificity or targeting is accomplished under these conditions. A working model that outlines our current view of PAN RNA lncRNA function is presented in Figure 1.
